# Shared decision-making in end-stage renal disease: a protocol for a multi-center study of a communication intervention to improve end-of-life care for dialysis patients

**DOI:** 10.1186/s12904-015-0027-x

**Published:** 2015-06-12

**Authors:** Nwamaka D. Eneanya, Sarah L. Goff, Talaya Martinez, Natalie Gutierrez, Jamie Klingensmith, John L. Griffith, Casey Garvey, Jenny Kitsen, Michael J. Germain, Lisa Marr, Joan Berzoff, Mark Unruh, Lewis M. Cohen

**Affiliations:** Division of Nephrology, Department of Internal Medicine, Massachusetts General Hospital, Harvard Medical School, Boston, MA USA; Division of General Medicine, Department of Internal Medicine, Baystate Medical Center, Springfield, MA USA; Center for Quality of Care Research, Baystate Medical Center, Springfield, MA USA; Division of Nephrology, Department of Internal Medicine, University of New Mexico, Albuquerque, NM USA; Department of Psychiatry, Baystate Medical Center, Springfield, MA USA; Bouve College of Health Sciences, Northeastern University, Boston, MA USA; School of Nursing, Bouve College of Health Sciences, Northeastern University, Boston, MA USA; Kitsen and Associates, Chester, CT USA; Division of Nephrology, Department of Internal Medicine, Baystate Medical Center, Springfield, MA USA; Division of Geriatrics and Palliative Medicine, Department of Internal Medicine, University of New Mexico, Albuquerque, NM USA; Smith College School for Social Work, Smith College, Northampton, MA USA

**Keywords:** Dialysis, End-of-life, Advance care planning, Preferences, Palliative nephrology

## Abstract

**Background:**

End-stage renal disease carries a prognosis similar to cancer yet only 20 % of end-stage renal disease patients are referred to hospice. Furthermore, conversations between dialysis team members and patients about end-of-life planning are uncommon. Lack of provider training about how to communicate prognostic data may contribute to the limited number of end-of-life care discussions that take place with this chronically ill population. In this study, we will test the Shared Decision-Making Renal Supportive Care communication intervention to systematically elicit patient and caretaker preferences for end-of-life care so that care concordant with patients’ goals can be provided.

**Methods/design:**

This multi-center study will deploy an intervention to improve end-of-life communication for hemodialysis patients who are at high risk of death in the ensuing six months. The intervention will be carried out as a prospective cohort with a retrospective cohort serving as the comparison group. Patients will be recruited from 16 dialysis units associated with two large academic centers in Springfield, Massachusetts and Albuquerque, New Mexico. Critical input from patient advisory boards, a stakeholder panel, and initial qualitative analysis of patient and caretaker experiences with advance care planning have informed the communication intervention. Rigorous communication training for hemodialysis social workers and providers will ensure that standardized study procedures are performed at each dialysis unit. Nephrologists and social workers will communicate prognosis and provide advance care planning in face-to-face encounters with patients and families using a social work-centered algorithm. Study outcomes including frequency and timing of hospice referrals, patient and caretaker satisfaction, quality of end-of-life discussions, and quality of death will be assessed over an 18 month period.

**Discussion:**

The Shared Decision-Making Renal Supportive Care Communication intervention intends to improve discussions about prognosis and end-of-life care with end-stage renal disease patients. We anticipate that the intervention will help guide hemodialysis staff and providers to effectively participate in advance care planning for patients and caretakers to establish preferences and goals at the end of life.

**Trial registration:**

NCT02405312

## Background

More than 600,000 patients in the United States have end-stage renal disease (ESRD), with approximately 450,000 patients undergoing hemodialysis (HD) [1]. Mortality rates for hemodialysis patients are several fold greater than those in the general population and disproportionately higher rates occur in patients over the age of 75. This elderly population also represents the fastest growing segment of the chronic kidney disease (CKD) population in the United States [1, 2]. Wong and associates recently reported that 49 % of elderly long-term HD patients spent time in an intensive care unit (ICU) in their final month of life compared with 24 % of cancer patients – suggesting that patients with ESRD experience higher intensity of care at the end of life compared to other Medicare beneficiaries with life-limiting illnesses. This is consistent with the finding that only 20 % of dying dialysis patients currently receives hospice care while 55 % of people dying from cancer receive hospice services [3, 4].

Most ESRD patients want to learn about end-of-life (EOL) issues, such as treatment options (including withdrawal from dialysis), and the availability of hospice services [5, 6]. Discussing prognosis is a key step in EOL planning, but occurs infrequently or late in the dying process among patients with ESRD. In two studies, 95 % and 97 % of patients with ESRD preferred to be given life-expectancy information—even if their prognosis was poor [5, 7]. Furthermore, patients specifically want their physicians to disclose this information without prompting [5]. A validated prognostic tool exists for dialysis patients [8], yet both uncertainty regarding individual prognosis and a lack of training about how to communicate prognostic data has limited EOL discussions between nephrologists and patients.

In this study, we will test the Shared Decision-Making Renal Supportive Care (SDM-RSC) communication intervention to determine its impact on EOL care and outcomes. The intervention has been designed to systematically elicit patient preferences for EOL care so that preference-concordant care can be provided. The study outcomes will include the frequency and timing of hospice referrals, location of death and the quality of EOL planning discussions. Patient and caregiver satisfaction and assessment of the quality of death will also be assessed. The study will contribute to our understanding of how communication regarding prognosis and EOL preferences impacts EOL outcomes for ESRD patients and families.

## Methods/design

### Study design

This multi-center study is designed to test the effectiveness of an intervention to improve EOL communication for hemodialysis patients who are at high risk of death in the ensuing six months. The intervention will be carried out using a prospective cohort with a retrospective cohort serving as the comparison group. In this multi-modal intervention, nephrologists and social workers will communicate prognosis and provide EOL planning in face-to-face encounters with patients and families using a social work-centered algorithm. Follow-up sessions with the social worker will take place monthly following the initial discussion to provide further support, education, information, and referral to resources such as hospice.

### Study sites

Patients will be recruited from 16 dialysis units associated with two large academic centers; eight units are affiliated with Baystate Medical Center in Springfield, Massachusetts and eight are affiliated with the University of New Mexico in Albuquerque, New Mexico. Aggregate demographic data for all dialysis units are displayed in Table 1. The institutional review boards at Baystate Medical Center and University of New Mexico approved this study.

**Table 1:** Aggregate dialysis clinic data

	Baystate Medical Center	University of New Mexico
Age (years)	64.9	60.6
Female	41.0 %	45.1 %
Black	13.9 %	3.0 %
Hispanic	9.5 %	52.3 %
Deaths (per 100 pt/year)	25.5	17.7
Diabetes mellitus	36.5 %	64.9 %
Average number of comorbid diseases	4.2	3.6
Percent of deaths with hospice	26.1 %	25.9 %

### Participants

Prognosis will be determined for all patients attending the study dialysis clinics by relying on a previously developed prognostic tool [8]. Predictors of six-month mortality in this instrument are age, serum albumin, absence/presence of dementia, absence/presence of peripheral vascular disease and a modified Surprise Question (SQ). The SQ asks nephrologists “Would you be surprised if this patient died during the next six months?” Hemodialysis patients who are 1) English or Spanish-speaking, 2) ≥ 18 years of age, and 3) who fall into the highest quartile of predicted mortality risk are eligible for participation in this study. Exclusion criteria include 1) having a diagnosis of a severe psychiatric disorder (i.e. schizophrenia, bipolar disorder, etc., warranting hospitalization in the past month), 2) expectation of native kidney function recovery, 3) scheduled for living donor kidney transplant, 4) history of poor adherence to hemodialysis treatments (i.e. missing ≥ 4 treatments in the last month), and 5) exclusion by the primary nephrologist or social worker due to risk of harm. Children < 18 years of age will not be eligible for study participation as the physical factors related to ESRD are not directly comparable to those of adults. Patients must be willing and able to sign the consent form. If lacking capacity to meaningfully participate in medical decisions, patients must have a surrogate who is willing to sign the informed consent.

### Intervention development

#### Qualitative study

We conducted in-depth interviews with patients and families at both study locations. These interviews were intended to expand our knowledge regarding ESRD patient and family perspectives on EOL planning and to provide formative data for development of the intervention. Patient and family members’ prior experiences with preferences for EOL care were assessed. The qualitative analysis confirmed that many patients want to hear about prognosis and options for EOL care and that they would like their nephrologists to be involved in these discussions. Some patients also commented on developing relationships with their social workers as they assisted with care coordination and provided legal forms [9].

#### Stakeholder panel

This panel is composed of seven patient/family advocates, administrators, and interdisciplinary clinician-researchers. The former director of the ESRD Network of New England chairs the panel. Members include representatives of the largest for-profit and largest non-profit proprietary dialysis chains in the US, an experienced dialysis nurse, leaders of two community hospice programs and the director of the Patient and Family Council at one of the main study academic centers. The panel provides critical input for all steps of the study, assists with the development and implementation of the intervention, and will facilitate national dissemination.

#### Patient advisory board

Each study site has a patient advisory board with a combined total of 13 members. These boards are comprised of patients from participating dialysis units. The Baystate Medical Center board is chaired by a former chair of the Baystate Patient Advocacy Board who has experience with a family member undergoing dialysis. A palliative medicine physician chairs the University of New Mexico board. The patient advisory boards play a similar role to the stakeholder panel, but provide patients’ perspectives on decisions regarding study design, implementation, and ultimately dissemination of results.

#### Intervention training

Social workers and nephrologists have been trained to carry out the SDM-RSC intervention. Training included introductory lectures to all of the nephrologists and social workers caring for patients at the 16 intervention clinics. These 60-min training sessions consisted of a study overview, review of previous research, and explanation of the intervention. Trainees also will review a training tape produced by the study investigators and members of the stakeholder panel and patient advisory board (https://www.youtube.com/watch?v=uzBE7uz3cm4). The video models a patient-family-staff prognostic meeting [10–12], and builds on the approaches used in both clinical practice and clinical trials to improve communication with oncology patients [12–16]. Didactic resources will include a bibliography of recommended literature and the Renal Physicians Association (RPA) Guidelines for the Initiation and Discontinuation of Dialysis [17].

The dialysis social workers will be the principle facilitators of EOL communication between staff, patients, and families. Accordingly, they underwent additional training in an intensive one-day, interactive teleconference sponsored by Baystate Medical Center and Smith College School for Social Work [18]. The course trained the social workers to facilitate EOL goal-setting and prognostic communication during the SDM-RSC intervention sessions. Social workers in both sites will also engage in an eight-hour videoconference training on communicating bad news, ethical and cultural issues, and taking leadership on the team. They will continue to meet through telephonic conferences to discuss clinical and leadership matters. The introductory courses, training video and didactic resources will be provided in binders and uploaded to iPads. These will be distributed to the social workers as additional resources.

#### Evaluation of intervention fidelity

Standardized training and monitoring are key strategies to enhance the reliability and validity of the SDM-RSC intervention [19]. Treatment fidelity of the intervention will be enhanced by use of a checklist for social workers and nephrologists to use during discussions with patients [18]. There will be regular monitoring to assess for any variability in provider skills. A random selection of 10 % of the initial staff-patient-family meetings will be audiotaped with patients’ consent. The recordings will be reviewed by study co-investigators to ascertain adherence to the protocol. [20]

### Recruitment

The investigators will use a multi-faceted approach to recruitment. The staff of the dialysis units, including nurses, technicians, physicians, and social workers will be educated about the study rationale and scientific importance of the study. Special attention will be accorded to the recruitment of eligible minority patients to ensure diversity of the sample cohort.

### Study procedures

**Fig. 1:**
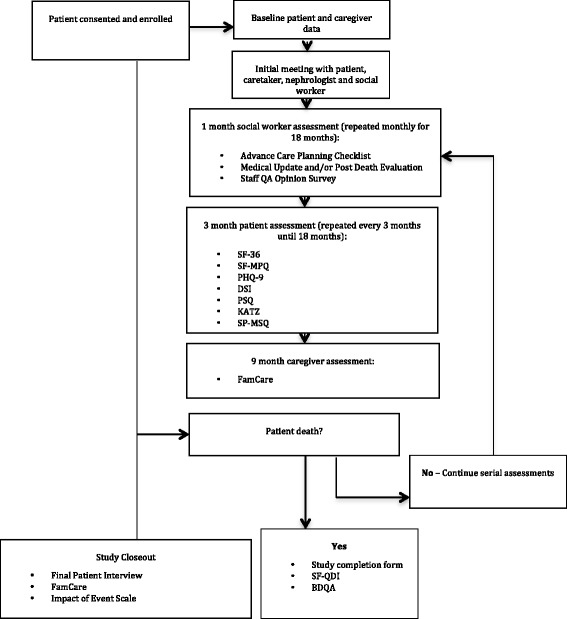
Study Procedures. Abbreviations: SF-36 = Short Form – 36, SF-MPQ = Short Form McGill Pain Questionnaire, PHQ-9 = The Patient Health Questionnaire, DSI = Dialysis Symptoms Index, PSQ = Patient Satisfaction Questionnaire, KATZ = Katz Index of Independence in Activities of Daily Living, SP-MSQ = Cognition Short Portable Mental Status Questionnaire, SF-QDI = Short Family Quality of Death Interview, BQDA = Baystate Quality of Dying Assessment

We will collect baseline data for patients and caregivers (see Table 2) at the time of enrollment. The initial patient and caretaker discussion will occur with the dialysis nephrologist and social worker. Patient and caretaker preferences for prognostic and EOL discussions will be broached in this meeting with dedicated follow-up by the social worker within 24 to 48 hours. Further data collection will take place at distinct time intervals (see Fig. 1). These will be assessed through interviews with the study coordinators and dialysis social workers. Additional data about hospice referrals will be collected from administrative data. Patient flow will be tracked to capture those who are lost to follow-up and reasons for dropout.

**Table 2:** Baseline assessment data

Patient level characteristics	Age, sex, race, ethnicity, marital status, type of insurance, level of formal education, employment, cause of ESRD, dialysis access, dialysis duration, comorbidities, income, household size, social support, health behaviors, religious affiliation, history of renal transplant, routine laboratories, dialysis clearance, functional status, health literacy, cognitive impairment, treatment adherence, and advanced directives
Caregiver characteristics	Age, sex, race, ethnicity, and relationship to the participant

### Outcomes

The primary outcome for this intervention will be the timing and percentage of hospice referrals. This will be determined by administrative databases, as well as by interviewing bereaved family members and the dialysis staff. A secondary EOL outcome is the location of participant death (i.e., home, hospital, ICU, or nursing home) ascertained through the interviews with bereaved families and the dialysis social workers who cared for the patient prior to death. An additional secondary EOL outcome is completion of advanced directive documents. Caregivers will also be asked to respond to questions about the quality of dying. Primary and secondary outcomes are summarized in Table 3.

**Table 3:** Study outcomes

Study aims	Primary Outcome	Secondary Outcomes
EOL outcomes	Hospice Use	Location of death, Presence of advanced directives
Patient and caregiver-reported outcomes	-	Depressive symptoms, Caregiver satisfaction, Quality of life, Pain symptoms, Caregiver distress, Quality of dying

To determine patient and caregiver-reported outcomes, instruments were selected to measure health-related quality of life (HRQOL), pain, depression symptoms, satisfaction with care, and caregiver distress (see Table 4) [21–26].

**Table 4:** SDM-RSC health-related quality of life instruments

Questionnaire	Items	Domain
*Patient*		
Cognition Short Portable Mental Status Questionnaire	10	Mental well-being
Katz Index of Independence in Activities of Daily Living	6	Activities of daily living
Rand Short-Form 36	36	Physical and mental well-being
Short Form McGill Pain Questionnaire	15	Sensory and affective pain
Patient Health Questionnaire- 9	9	Depressive symptoms
Dialysis Symptom Index	30	Physical/emotional symptoms
Patient Satisfaction	23	Satisfaction with care
*Caregiver*		
FAMCARE Scale	20	Satisfaction with care
Baystate Quality of Dying Assessment	5	Quality of patient death
Impact of Event Scale	15	Caregiver distress

### Analysis

Preliminary analyses will investigate differences in patient outcomes between the intervention and control periods. Initial analyses will also investigate trends in hospice usage during the two years of retrospective data collected for the control cohort. Smooth plots and generalized additive models with hospice use as the binary outcome and date (measured as days from December 2009) as the independent predictor will be used to model potential changes over time in the hospice usage rate. If statistically significant non-linear associations are found, then change over time will be modeled using piecewise linear time variables. The extent of clustering within the dialysis units during the control periods will be estimated by the intra-class correlation coefficient. The HRQOL and quality of dying measures will be summarized and compared across the 16 study sites. Analyses will be done in SAS (version 9.3 or higher) with a nominal type I error rate of 0.05.

#### Sample size and power

Over the 12 months of intervention enrollment we will screen approximately 1048 patients at the 16 participating sites, of which 25 % (n = 262) will be “highest risk,” as determined by our validated model. We anticipate that 80 % of these high risk patients will enroll in the study and further expect that half of these patients will die during 18 months of follow-up, yielding at least 105 patients available for analysis. Hence, our sample size will be adequate to account for a 5 % loss of data in follow-up due to withdrawal from the study and the loss of power in accounting for unit or site variation.

## Discussion

The SDM-RSC intends to improve discussions about prognosis and EOL care with ESRD patients. We anticipate that the intervention will encourage advanced care planning and help patients establish EOL care preferences and goals. This study will examine whether the intervention affects use of hospice services and whether it influences patient outcomes such as hospitalizations, location of death, quality of life, caregiver distress, and quality of death. The intervention will be tested in diverse environments and geographic settings.

This is the first multi-center, prospective study designed to test the hypothesis that EOL treatment can be improved by identifying ESRD patients at high risk for death and providing a systematic approach to discussing prognosis and EOL preferences. The study is especially novel as it brings patients and stakeholders together with leading clinician-researchers who are interested in examining the integration of palliative medicine into the practice of nephrology. The primary investigators have previously conducted research involving severely ill and elderly ESRD patients [27–32]. The study’s interdisciplinary team contributes additional expertise in palliative care, communication, bioethics, psychiatry, and social work education [17, 33]. Previous investigations regarding EOL care have largely featured small observational studies or retrospective analyses [4, 5, 34]. For instance, Schmidt *et al*. analyzed the deaths of 65 dialysis patients in a single center hemodialysis unit over a five-year period [34]. Although patients who were approached to discuss dialysis withdrawal were less likely to die in the hospital and more likely to use hospice, there was not a consistent approach as to selection of patients or timing of discussions. As our investigation targets patients at the highest risk of dying, we will be investigating communication between providers, patients and caretakers during the most challenging emotional and physical circumstances. Furthermore, previous studies that have featured patient surveys and interviews to elicit preferences and knowledge about EOL have typically not incorporated family or caretaker beliefs in the interventions [5, 35]. Use of qualitative data to develop our intervention and use of interdisciplinary interventional teams among diverse patient populations are innovative elements of this study. Furthermore, reliance on patient advisory boards and a stakeholder panel to shape the study design and implement the intervention represent a unique approach for improving delivery of prognostic information and elicitation of EOL care preferences. These are consistent with recommendations by the RPA for EOL care [17] and the Institute of Medicine’s advocacy of patient-centered healthcare [31, 36–39]. We believe that our communication intervention has relevance to not only ESRD but also other high-mortality disorders [13, 14, 20, 40–42].

This study will help establish whether delivery of prognostic information, encouragement of advance directives, and sensitive discussion of terminal treatment options, can meaningfully alter the last portion of a patient’s life. The proposed investigation may also gently confront the issue of treatment overuse, as the HD literature has begun to question the circumstances in which potential for harm may exceed possible benefit [17, 43]. In summary, if the SDM-RSC intervention is successful, it has the potential to increase hospice use and significantly improve the quality of life for patients with severe kidney disease.

## Abbreviations

ESRD: End-stage renal disease
HD: Hemodialysis
CKD: Chronic kidney disease
ICU: Intensive care unit
EOL: End-of-life
SDM-RSC: Shared Decision-Making Renal Supportive Care
SQ: Surprise question
RPA: Renal Physicians Association
HRQOL: Health-related quality of life
